# Progress in Surgery

**Published:** 1897-12-18

**Authors:** 


### Progress in Surgery.

### GENERAL SURGERY.

**Antiseptics.**—Zeroform (Bismuth tribromophenol) has received further practical investigation at the hands of Dr. Goliner[1] and Dr. Josef Grünfeld.[2] The latter states that this drug not only possesses a powerful anti-bacterial action, but by combining with toxins and ptomaines renders them harmless, setting free bismuth, which makes insoluble compounds with these poisons; it has marked drying and astringent properties; it is a deodorant, and therefore particularly useful for wound cavities about the digestive or urinary tract; it does not irritate wounds or soft parts; it relieves pain through its contained bismuth and partly through the protective covering which it forms; it is non-poisonous; it stimulates granulation-tissue and skin formation; and, lastly, it is hæmostatic. Since the publication of Credé's paper on "Silver and the Silver Salts in their Surgical and Bacteriological Relationships," referred to in The Hospital of September 4th, Dr. A. Tilger[3] has used the citrate of silver in various cases. His observations entirely agree with the statments of Credé, and he believes that the silver treatment of wounds will, in private practice, replace the aseptic method, which can hardly be carried out by the general practitioner. He has used the citrate in some 50 to 60 cases of injury and minor surgical procedure as they occurred in general practice. The results have been such as to lead him to employ it exclusively in his surgical work. It is absolutely non-poisonous and non-irritating; it has powerful antiseptic and bactericidal properties, which are exercised on the deeper tissues also; it has a favourable healing and cicatrising effect on wounds; and, finally, it is readily applied and is cheap, since only the thinnest possible layer need be dusted on. Tilger does not hesitate to close even non-linear injuries with plaster after powdering them with the citrate. Such wounds heal quickly and soundly under a dry scab, and with a very few changes of dressing. Dr. A. E. Gallant[4] discourses upon the value of castor oil and balsam of Peru as a surgical dressing which prevents retention and favours drainage and healing. He has used it in many cases with excellent results. There was no scabbing, no redness, no eczema, no adhesion of the dressing; epithelial growth went on rapidly; there was no irritation, pain, fever, or infection. As to the original use of the balsam-oil dressings Dr. W. W. Van Arsdale[5] said that he had been driven to its use through necessity rather than through prethought. Being trained in the aseptic and antiseptic schools, it was not from choice that he had treated the first cases in this way in dispensary work, where it was impossible to carry out the details of antiseptic or even aseptic treatment. Wherever drainage had to be substituted for asepsis he most usually recommended the dressing. In times of war it would be of special benefit, and ought, he thought, to take the place of the iodoform gauze which every soldier in the Germany army had to carry. The latter was calculated to shut in secretion and favour sepsis, especially if there were infection, as there was likely to be. The balsam oil keeps the wound dry and yet moist. The treatment of burns by picric acid, which has been drawn attention to by M. M. Thiéry, Papazoglou, Filleul, Gaucher, Leredde, &c., has been supplemented by M. Debuchy's[6] investigations respecting the solubility of this acid in the principal vehicles for surgical work. Mr. Mills[7] has also recently read a paper on picric acid as a primary dressing in the same injuries. Whether in superficial or serious burns the objects aimed at are: to relieve pain, to prevent sepsis, to allay inflammation, to prevent the spread of inflammation to devitalized tissue, to promote rapid separation of sloughs by dry gangrene; to encourage healthy granulations. He cleanses the parts with weak carbolic lotion, pricks the blisters, and applies surgical lint soaked in a saturated solution of picric acid (picric acid, ʒijs.; absolute alcohol, ℥iii.; water to ℥xl.) wool, and a many-tailed bandage. Chloroform was given, especially to children. Subsequent dressings, generally twice a week, are guided by the discharge, temperature, &c., care being taken not to tear away adherent lint. Mr. Miles has treated 100 cases in Leith Hospital. The advantages are simplicity, painlessness, asepsis, small amount of discharge, infrequent dressings, astringent action in preventing inflammation, promotion of growth of epithelium, rapid separation of sloughs, absence of poisoning symptoms, and economy in dressings. The disadvantages were the staining of the hands and bedclothes. Formalin is the most powerful bactericidal agent known, according to Dr. P. Rosenberg's[8] experiments with this substance and its derivatives, known under the names of holzin and steriform, which are obtained by dissolving it in methylic alcohol. Holzin is an aqueous solution, which rapidly evaporates; steriform is a solid substance. These experiments showed that formaldehyde is the most powerful disinfecting agent for rooms, wearing apparel, and furniture; while taken internally it is a non-toxic—a 1 in 100,000 solution of formaldehyde prevents prolification of microbes, and more concentrated solutions are bactericidal. In several patients suffering from phthisical hyperpyrexia the fever appeared to subside under the administration of formaldehyde, and their expectoration changed. Holzin may also be utilised for sterilising surgical instruments by placing them for fifteen minutes in this liquid. Steriform (the derivative of formaldehyde) is eliminated by the urine, which is thus rendered sterile. Dr. D. Semple[8] gives a note on the bacteriological conditions present in ulcers treated by oxygen gas (Stoker's method), and the practical results of a few cases, all of which were in favour of this treatment. The oxygen possibly acts in one or more of the following ways: Diminution of irritation, direct stimulation without irritation, oxidation of the toxins produced by micro-organisms in the surface of the ulcer; and, as stated by Mr. Stoker, the selective power in its action on micro-organisms present in the ulcer, encouraging staphylococci, which then outgrow the bacilli. Mr. A. E. Mayland[10] records brief extracts of four cases, showing the great potency of pure carbolic acid, when freely applied, of rendering rapidly aseptic all the parts of an acutely septic wound. Some ounces of the pure acid were brushed over the raw and freely-exposed surfaces in some cases of acute traumatic gangrene, and in each instance improvement set in immediately. There appears to be no pain from the application, except so far as is entailed in the mechanical manipulation of the wound and its surfaces. When sloughs exist, these should be practically soaked in the acid. On living tissues the acid has no deleterious effect, but it excoriates the skin and causes pain. Some interesting and valuable particulars regarding the relative value, preparation, and composition of some aseptic and antiseptic dressings and accessories, as used by the surgeon, have been recently published by M. L. Adrian[11] in a series of articles. As heat cannot be applied in the required disinfection and aseptic condition of the hands of the surgeon and assistants, this affords yet a weak spot in aseptic work, and numerous attempts have been made to gain both certainty and simplicity in this direction. Dr. Robt. E. Weir[12] has come to the conclusion that, though alcohol will not accomplish all, or nearly all, that Reinecke and others claim for it, it is, nevertheless, a valuable, if not the most valuable, agent for disinfection of the hands. For the same purpose chlorine is equally efficient. The method of employing the latter for hand disinfection is to put a large pinch of household chlorinated lime or bleaching powder in the palm of the hand, and on it is placed a crystal of carbonate of sodium (common washing soda), about 1 in. wide; a little water is added, and a thick cream forms, which is well rubbed for three or five minutes into the hands and arms. The paste is washed off in sterile water. Weir concludes that for hand disinfection solutions of corrosive sublimate are unreliable, that it is far best effected, and in the order named, by the use of nascent chlorine, alcohol, or potassium permanganate; that chlorine is satisfactorily evoked by the conjoined use of moistened commercial choride of lime and crystallised carbonate of soda; that of these three procedures, the chlorine treatment is the least hurtful to the hands, alcohol the most trying. Professor J. Mukulicz[13] says that it is an open secret that the results of asepsis are scarcely any improvement over antisepsis, and finds from a long series of tests that it is impossible to render the hands perfectly aseptic. He has therefore commenced to wear gloves at his work. He uses cheap gloves, sold as "fine servants' gloves" and waiters' gloves, which he buys in Breslau for 65 cents a dozen. They are linen or cotton and can be washed and boiled, and used over and over again. He first disinfects his hands as carefully as possible with the alcohol sublimate method, and then draws on the gloves. They do not interfere with the operation, and even allow a firmer grip of the sutures and tissues. If the operation is short and aseptic, one pair of gloves is sufficient; but if not, two or three changes are required at different steps of the operation. The assistants also wear the gloves, and change at the same time.

[1] Reichs Medicinal Anzeiger, No. 7, s. 100. [2] Wiener Medicinische Blätter, 1897, No. 1—3, and Amer. Jour. of the Med. Sciences, June, 1897. [3] Munchener Medicinsche Wochenschrift, Feb. 9, 1897, and Charlotte Medical Jour., April, 1897, p. 474. [4] N.Y. Med. Record, March 27, 1897, p 457. [5] Ibid., p. 458. [6] Les Nouveaux Remédes, Aug. 24, 1897, p. 481. [7] Brit. Med. Jour., July 17, 1897. [8] Medical Week, April 23, 1897, p. 189. [9] Lancet, May 29, 1897, p. 1,465. [10] Brit. Medical Jour., June 12, 1897, p. 1 475. [11] Les Nouveaux Remédes, Aug. 8 and Aug. 24, 1897, &c. [12] New York Med. Record, April 3, 1897, p. 469. [13] Journal of the Amer. Med. Association and Clinical Journal, Sept. 8, 1897, p. 319

